# Cutaneous Delivery of Cosmeceutical Peptides Enhanced by Picosecond- and Nanosecond-Domain Nd:YAG Lasers with Quick Recovery of the Skin Barrier Function: Comparison with Microsecond-Domain Ablative Lasers

**DOI:** 10.3390/pharmaceutics14020450

**Published:** 2022-02-19

**Authors:** Woan-Ruoh Lee, Chien-Yu Hsiao, Zi-Yu Chang, Pei-Wen Wang, Ibrahim A. Aljuffali, Jie-Yu Lin, Jia-You Fang

**Affiliations:** 1 Graduate Institute of Medical Sciences, Taipei Medical University, Taipei 110, Taiwan; wrlee@tmu.edu.tw; 2 Department of Dermatology, Taipei Medical University Shuang Ho Hospital, New Taipei City 234, Taiwan; 3 Department of Nutrition and Health Sciences, Chang Gung University of Science and Technology, Kweishan, Taoyuan 333, Taiwan; nulycopene@gmail.com; 4 Research Center for Food and Cosmetic Safety and Research Center for Chinese Herbal Medicine, Chang Gung University of Science and Technology, Kweishan, Taoyuan 333, Taiwan; 5 Aesthetic Medical Center, Department of Dermatology, Chang Gung Memorial Hospital, Kweishan, Taoyuan 333, Taiwan; 6 Department of Traditional Chinese Medicine, Chang Gung Memorial Hospital, Keelung 204, Taiwan; changzhi887@gmail.com; 7 Institute of Traditional Medicine, School of Medicine, National Yang Ming Chiao Tung University, Taipei 112, Taiwan; 8 Department of Medical Research, China Medical University Hospital, China Medical University, Taichung 404, Taiwan; pwwang5105@hotmail.com; 9 Department of Pharmaceutics, College of Pharmacy, King Saud University, Riyadh 11362, Saudi Arabia; ialjuffali@ksu.edu.sa; 10 Pharmaceutics Laboratory, Graduate Institute of Natural Products, Chang Gung University, Kweishan, Taoyuan 333, Taiwan; d000011794@cgu.edu.tw; 11 Department of Anesthesiology, Chang Gung Memorial Hospital, Kweishan, Taoyuan 333, Taiwan

**Keywords:** Nd:YAG laser, CO_2_ laser, Er:YAG laser, laser-assisted delivery, skin absorption, cosmeceutical peptide

## Abstract

Picosecond or nanosecond-domain non-ablative lasers generate faster photothermal effects and cause less injury than microsecond lasers. In this study, we investigated the enhancing effect of 1064 nm picosecond- and nanosecond-domain neodymium (Nd):yttrium–aluminum–garnet (YAG) lasers on the cutaneous delivery of cosmeceutical peptides. Microsecond-domain fractional ablative CO_2_ and fully ablative erbium (Er):YAG lasers were also used for comparison. In the Franz diffusion cell study, pig or mouse skin was treated with a laser before exposure to palmitoyl tripeptide (PT)-1, PT-38, and copper tripeptide (CT)-1 at a concentration of 150 μM. Psoriasiform, atopic dermatitis (AD)-like, and photoaged skins were also developed as permeation barriers. The non-ablative laser elicited the ultrastructural disruption of the stratum corneum and epidermal vacuolation. All laser modalities significantly increased the skin permeation of peptides in vitro. The non-ablative laser chiefly enhanced peptide delivery to the receptor compartment, whereas the ablative laser mainly increased the intracutaneous peptide deposition. The picosecond- and nanosecond-domain Nd:YAG lasers elevated the amount of PT-1 in the receptor up to 40- and 22-fold compared with untreated skin, respectively. Laser treatment promoted peptide delivery in barrier-deficient and inflamed skins, although this enhancement effect was less than that observed in healthy skin. Fluorescence microscopy indicated the capability of the non-ablative laser to deliver peptides to deeper skin strata. The ablative laser confined the peptide distribution in the epidermis. Confocal microscopy showed that peptides penetrated the skin along the microdots created by the fractional Nd:YAG and CO_2_ lasers. The skin barrier function determined by transepidermal water loss suggested quick recovery when using a nanosecond-domain laser (within 4 h). A longer period was needed for the skin treated with the fully ablative Er:YAG laser (76−84 h). Nanosecond non-ablative laser-facilitated peptide delivery may become an efficient and safe approach for cosmeceutical applications.

## 1. Introduction

Peptides are short chains of amino acids. Many peptides have been developed for cosmeceutical and anti-aging uses in the last two decades [1]. Cosmeceutical peptides demonstrate the bioactivities of collagen and elastin synthesis, fibroblast proliferation, cell migration, inflammation mitigation, and melanogenesis inhibition [2]. These bioactivities help improve skin tightness, elasticity, and firmness. Photoaging, wrinkling, and hyperpigmentation are also reduced [3]. These topical peptides for cosmeceutics have the advantages of easy synthesis, high stability, and minimal skin irritation [4]. However, topical peptides face problems in skin delivery and targeting due to the barrier function of the stratum corneum (SC) and the tight junction for permeation [5], lowering the translation to clinical application. To enhance the low diffusion of some permeants, physical techniques such as tape stripping, iontophoresis, sonophoresis, microneedles, radiofrequency, and lasers have been employed to improve skin delivery. The use of ablative lasers at low fluence is advantageous for peeling superficial SC and thereby promoting skin absorption. Laser treatment can selectively and precisely remove superficial skin in controlled and noncontact modes without the production of biohazardous waste [6]. Laser-assisted drug delivery has proven to be beneficial for treating melasma, actinic keratosis, skin cancers, vitiligo, and keloid scars in clinical practice [7,8].

CO_2_ and erbium (Er):yttrium–aluminum–garnet (YAG) lasers are ablative modalities commonly used for laser-assisted drug absorption. The wavelengths of CO_2_ and Er:YAG lasers are 10,600 and 2940 nm, respectively. Both wavelengths preferentially target water to ablate the water-containing epidermis. Compared with a fully ablative modality, the fractional resurfacing of the lasers creates arrays of microdots surrounded by intact skin that facilitate drug permeation with quicker healing. The untreated skin area serves as a reservoir of stem cells that can rapidly migrate into the injured skin for fast recovery [9]. In addition to ablative lasers, non-ablative fractional lasers generate a photomechanical shock wave that disrupts the epidermal structure for enhanced skin delivery. This enhancement is attributed to the vacuoles in the epidermis or the subepidermal clefting in the dermal–epidermal junction [10]. Non-ablative lasers preserve the integrity of the SC to avoid the risk of pigment change and bacterial infection [11]. Recently, compared with microsecond-domain lasers, 1064 nm picosecond- and nanosecond-domain neodymium (Nd):YAG lasers have been introduced to create fast photothermal reactions and cause less skin impairment [12]. These lasers are used to treat tattoo, photoaging, and acne scarring in clinics [13]. To decrease thermal damage, the picosecond or nanosecond pulse duration is shorter than the thermal relaxation time of the skin tissue. A reduction in thermal coagulation is advantageous for drug transport because of the role of coagulated tissues in retarding diffusion [14]. Lasers with shorter pulse times usually reveal stronger photoacoustic effects with lower risks of side effects or pain [15].

**Figure 1:**
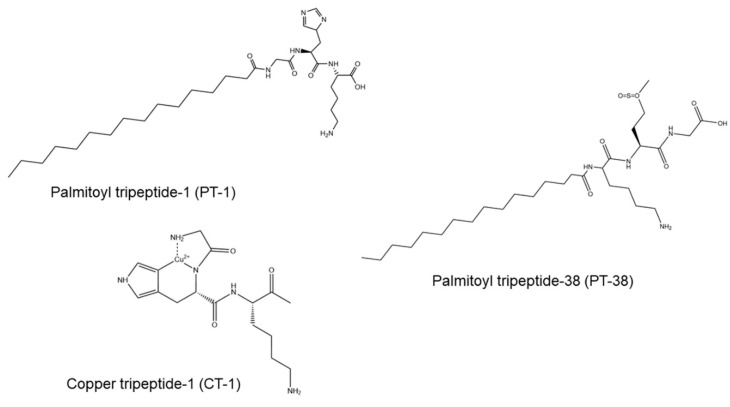
The chemical structures of the cosmeceutical peptides PT-1, PT-38, and CT-1.

In this study, we aimed to facilitate the topical delivery of cosmeceutical peptides by picosecond- and nanosecond-domain Nd:YAG lasers with minimal invasiveness of the skin. Peptide absorption enhancement by non-ablative lasers was compared with that by microsecond-domain ablative lasers, including a fractional ablative CO_2_ modality and a fully ablative Er:YAG modality. In this study, palmitoyl tripeptide (PT)-1, PT-38, and copper tripeptide (CT)-1 with different amino acid sequences, lipophilicities, and molecular sizes were chosen as model permeants (Figure 1). We first examined the appearance and structure of the laser-treated skin. Then, laser-mediated peptide permeation was assessed by an in vitro Franz cell platform. Barrier-deficient skin and inflamed skin were also utilized as permeation barriers to check the capability of laser-assisted peptide delivery to diseased skin. Finally, in vivo peptide absorption and skin barrier function recovery were evaluated in a mouse model.

## 2. Materials and Methods

### 2.1. Materials

All the peptides conjugated with fluorescein isothiocyanate (FITC) at the C-terminal were supplied by Biotools (New Taipei City, Taiwan) with purity > 97%. Imiquimod cream (Aldara) was provided by 3M Healthcare (Leicestershire, UK). The cyanoacrylate superglue was purchased from 3M (St. Paul, MN, USA). The hematoxylin and eosin (H&E) staining kit was supplied by Abcam (Cambridge, UK).

### 2.2. In Silico Molecular Modeling

The physicochemical features of the peptides were estimated using a Discovery Studio 4.1 workstation (Accelrys, San Diego, CA, USA). These features included the molecular volume (MV), theoretical oil/water partition coefficient (Alog *P*), hydrogen bond acceptor number, hydrogen bond donor number, and total polarity surface.

### 2.3. Laser Devices

A 1064 nm Nd:YAG fractional laser (Picoplus, Lutronic, Goyang, Korea) with pulse durations of 450 psec and 2 nsec was used in this study. The laser fluence was set at 1.4 J/cm^2^. We estimated a single pulse of the laser comprising 9 × 9 microbeams arranged in a 7.4 × 7.4 mm^2^ square zone. Each microbeam irradiated a spot with a diameter of 100 μm. The fractional CO_2_ laser (Mosaic eCO_2_, Lutronic) used a 10,600 nm wavelength with a pulse duration of 90 μs. The laser irradiated a fluence of 2 mJ over a scanning region of 14 × 14 mm. A microscopic thermal zone (MTZ) with a diameter of 300 μm was generated using 20 × 20 microbeams over 1 × 1 cm, and a fully ablative Er:YAG laser (Contour, Sciton, Palo Alto, CA, USA) with a wavelength of 2940 nm was employed for comparison. A square scanning area of 15 × 15 mm was utilized to irradiate 16 spots with a diameter of 3.5 mm and a 10% overlap. The fluence and pulse duration of the Er:YAG laser were 5 J/cm^2^ and 100 μs, respectively.

### 2.4. Animals

One-week-old Duroc–Landrace crossbred pigs were supplied by Pigmodel Animal Technology (Miaoli, Taiwan), and eight-week-old Balb/c and nude mice were purchased from the National Laboratory Animal Center (Taipei, Taiwan). All the animal experiments were approved by the Institutional Animal Care and Use Committee of Chang Gung University and complied with Directive 86/109/EEC of the European Commission.

### 2.5. Laser-Irradiated Skin Imaging

Dorsal skin was excised from the pigs and exposed to the lasers to visualize the skin surface and morphology. The microscopic appearance of the skin surface was observed using a handheld digital magnifier (Mini Scope-V, M&T Optics, Taipei, Taiwan) and an optical microscope (DMi8, Leica, Wetzlar, Germany). The skin surface was also monitored by scanning microscopy (SEM, Leica TCS SP8 X) at a larger magnification (250×). The skin was immersed in formalin and embedded in paraffin wax for slicing at 5 μm thickness. The slice was stained with H&E to observe the skin histology under optical microscopy.

### 2.6. Induction of Inflamed Skin in Mice

Inflamed skin was induced in the mice to examine peptide permeation through diseased skin. Three forms of inflamed skin were induced, namely, psoriasis-like, atopic dermatitis (AD)-like, and photoaged skins. The psoriasiform plaque in the Balb/c mouse back was evoked based on the method described by van der Fits et al. [16]. The mice received a daily dose of 62.5 mg 5% imiquimod cream on a shaved dorsal skin region for five consecutive days. The mice were sacrificed on day six for further skin permeation experiments. Cutaneous sensitization for inducing AD-like skin was the same as in our previous study [17]. Briefly, the Balb/c mice were sensitized with ovalbumin (0.1 mL) at a dose of 1 mg/mL through an intraperitoneal injection every other day for 10 days. The fur on the back was removed and tape-stripped six times on day 8. Ovalbumin (1 mg/mL) in saline with a volume of 0.1 mL was poured onto a 1 × 1 cm sterile gauze and then topically administered on the dorsal region. The gauze was applied each day for seven days to induce an AD-like lesion. For the induction of photoaged skin, the backs of the nude mice were irradiated by UVA at 365 nm (Bio-Spectra, Vilber Lourmat, Collégien, France) with a spectral irradiance of 3 J/cm^2^ (Lin et al., 2018). The mouse backs were exposed to UVA once a day for five days.

### 2.7. Preparation of the Barrier-Deficient Skin

SC-stripped, delipidized, and deproteinized pigskins were prepared for the evaluation of cutaneous peptide delivery. An adhesive cellophane tape (3M Scotch) was applied to the excised pigskin and then removed 20 times to obtain tape-stripped skin. Delipidized skin was developed by incubating the skin surface with chloroform/methanol (2:1) for two hours and then washing it with water several times. Protein denaturation of the skin was achieved by incubating the skin surface in ethanol/water (2:3) for two hours [18].

### 2.8. In Vitro Peptide Permeation

A vertical Franz cell assembly (Chingfa Glass Company, Hsinchu, Taiwan) was employed for the assay of the in vitro peptide delivery. The thickness of the full-thickness pigskin used in this study was about 0.55 mm. The intact, inflamed, or barrier-deficient skins with or without laser treatment were mounted between the donor and receptor compartments with the SC facing the donor. Both the donor and receptor media contained a pH 7.4 citrate–phosphate buffer. The donor was filled with 0.5 mL of FITC-conjugated peptides (150 μM) in a buffer. The effective permeation region was 0.785 cm^2^. The stirring rate and temperature of the receptor medium (5 mL) were kept at 600 rpm and 37 °C, respectively. The donor temperature was maintained at room temperature (about 25 °C). Thus, the temperature of the excised skin surface could be kept at about 32 °C, mimicking human skin temperature. The 300 μL aliquot in the receptor compartment was withdrawn after 24 h. The skin was then removed to examine the intracutaneous deposition of topical peptides. The skin was extracted with 0.1 N HCl in a MagNA Lyser (Roche, Penzberg, Germany). The homogenate was centrifuged at 10,000× *g* for 10 min to obtain the supernatant solution. The peptide content in hair follicles was extracted by differential stripping and cyanoacrylate casting, as described previously [19]. In brief, the SC of the skin was stripped with cellophane tape 20 times, after which a drop of superglue was added to a glass slide and pressed onto the skin surface. The cyanoacrylate polymerized, and the slide was expelled with one quick movement after 5 min. The remaining superglue on the slide was scraped off and positioned in a test tube with methanol (2 mL). The tube was shaken for three hours. The methanol in the tube was vacuumed and then added to water for quantification detection of follicular peptide accumulation. The peptide amount in all the samples was quantified using a fluorescence spectrophotometer (F2500, Hitachi, Tokyo, Japan) at the excitation and emission wavelengths of 490 and 520 nm, respectively.

### 2.9. Peptide Distribution in the Skin

The peptide distribution was visualized using fluorescence microscopy (Leica DMi8) and confocal laser scanning microscopy (Leica TCS SP2), along with the vertical and horizontal views, respectively. The skin was removed from the Franz cell within 24 h after PT-1 (150 μM) application. After washing with water, the skin sample was sectioned in a cryostat microtome at a thickness of 5 μm and then mounted using glycerin and gelatin. The slice was visualized with a fluorescence microscope using a filter set at 450−490 nm and 515−565 nm for excitation and emission, respectively. For the horizontal view, the skin sample was positioned onto the stage plate of the confocal microscope. The skin thickness was scanned at 5 μm increments through the z-axis from the cutaneous surface. Imaging was taken by adding 15 fragments in both the two-dimensional (2D) and three-dimensional (3D) modes.

### 2.10. In Vivo Intracutaneous Peptide Deposition

A glass cylinder with a hollow region of 0.785 cm^2^ was fixed to the back of nude mice using superglue. An aliquot (0.2 mL) of PT-1 (150 μM) in a pH 7.4 buffer was pipetted into the cylinder. Within six hours after application, the animal was sacrificed to remove the treated skin. The peptide in the skin was extracted with a MagNA Lyser in the same manner as in the in vitro skin deposition examination.

### 2.11. In Vivo Skin Barrier Function Recovery

The recovery of the skin barrier function after laser treatment was monitored by the transepidermal water loss (TEWL). A Tewameter (TM300, Courage and Khazaka, Köln, Germany) was employed to determine the TEWL. The TEWL of the laser-irradiated nude mouse skin was estimated until the level was reduced to the baseline control. The temperature and relative humidity of the TEWL measurement environment were kept at 25 °C ± 1 °C and 75% ± 5%, respectively.

### 2.12. Statistical Analysis

Statistical differences between the data of different treatment groups were measured using the Kruskal–Wallis test, while the post hoc test to check for individual differences was performed using Dunn’s test. The significant levels of probability included 0.05, 0.01, and 0.001.

## 3. Results

### 3.1. Laser-Treated Skin Imaging

**Figure 2:**
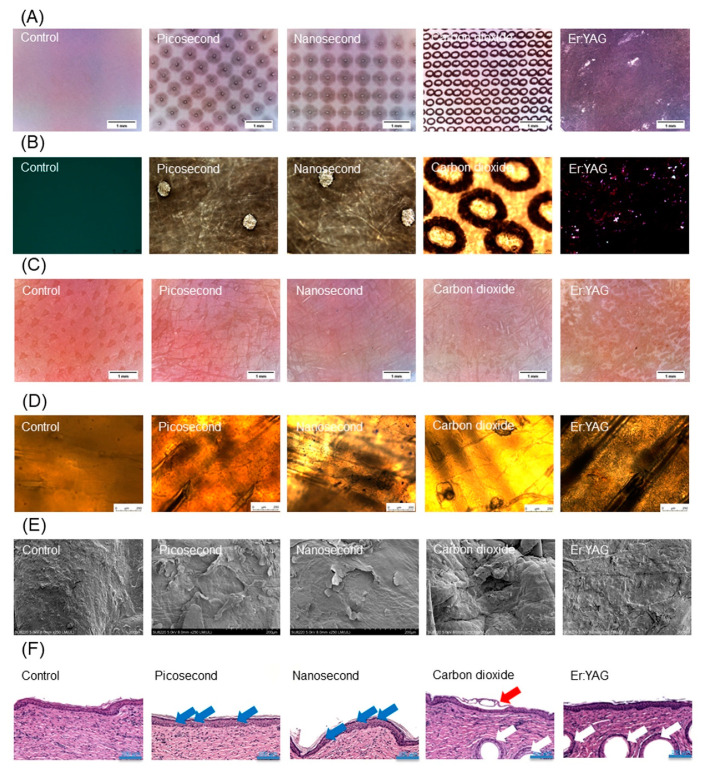
Images of the skin treated by picosecond-domain Nd:YAG, nanosecond-domain Nd:YAG, CO_2_, and Er:YAG laser. (**A**) Thermal paper imaging captured with a handheld digital magnifier; (**B**) thermal paper imaging captured by optical microscopy; (**C**) skin surface imaging captured with a handheld digital magnifier; (**D**) skin surface imaging captured by optical microscopy; (**E**) skin surface imaging captured by SEM; and (**F**) H&E staining of the skin observed by optical microscopy. The blue arrows in the image indicate vacuoles. The red arrows in the images indicate the micropores created by the CO_2_ laser. The white arrows in the images indicate hair follicles.

A preliminary examination of the laser treatment was performed using thermal paper as the model material. The microdot arrays generated by the picosecond- and nanosecond-domain Nd:YAG lasers were uniform and reproducible (Figure 2A). A thermal reaction was generated around the microdots after the Nd:YAG laser treatment. The CO_2_ laser produced arrays of dots with some thermal reactions on the thermal paper. The diameters of the microdots generated by the Nd:YAG modality and the CO_2_ modality were approximately 100 and 300 μm, respectively (Figure 2B). These sizes approximated the spot diameters of the microbeams produced by both lasers. The appearance of the pigskin surface treated by the picosecond- and nanosecond-domain Nd:YAG lasers demonstrated no change on the skin surface compared with the control group (Figure 2C). The pigskin exhibited fractionated zones of CO_2_ laser-induced tissue reaction on the cutaneous surface (Figure 2C, red arrow). Full ablation by the Er:YAG laser revealed an absence of furrows in the superficial layer. The skin surface was visualized by optical microscopy to provide close-up images under controlled light. No significant change in the skin surface was found after Nd:YAG laser exposure (Figure 2D). There was a clear puncture of micropores extending into the superficial epidermis by the CO_2_ laser. The resulting pore diameter generated by the CO_2_ laser was 100−150 μm, which was smaller than the microbeam size. There should be some thermal coagulation around the opening of the pore.

The SEM image of the control skin surface showed some roughness (Figure 2E). This roughness was lessened by the Nd:YAG laser application. A single pore produced by the fractional CO_2_ laser showed a diameter of approximately 150 μm, with protruded coagulation surrounding the pore. The size of the pore plus the thermal coagulation was approximately 300 μm. Complete ablation by the Er:YAG laser flattened the skin surface. H&E staining of the untreated skin displayed the characteristic layers of the intact SC, epidermis, and dermis (Figure 2F). The Nd:YAG laser with picosecond and nanosecond domains maintained an anatomically intact SC. Microscopic vacuolation was found in the upper epidermis after Nd:YAG laser treatment (Figure 2F, blue arrows). No significant disruption of the dermis was observed. We ascertained the presence of microscale passages developed by the fractional CO_2_ laser. The white circles in the dermis indicated hair follicles (Figure 2F, white arrows). The histological image depicted micropores penetrating the epidermis with some remnant SC layers (Figure 2F, red arrow). The Er:YAG laser peeled the SC and left the epidermis intact.

### 3.2. In Silico Molecular Modeling

The predicted physicochemical characteristics of the PT-1, PT-38, and CT-1 peptides were estimated using the Discovery Studio workstation to establish the relationship with skin delivery. The molecular weight (MW) of the peptides was between 344 and 605 Da, with the following sequence: PT-38 > PT-1 > CT-1 (Table 1). The MV of the peptides correlated well with the MW. The Alog *P* value indicated the lipophilic nature of PT-1 (2.3) and PT-38 (2.4), whereas CT-1 demonstrated a hydrophilic feature (−2.1). PT-38 showed the greatest number of total hydrogen bonds (acceptor bond + donor bond = 14), followed by CT-1 (13) and PT-1 (12). The total polarity surface of a molecule is defined as the surface sum of all atoms, primarily hydrogen, oxygen, and nitrogen. PT-38 demonstrated the highest total polarity surface level.

### 3.3. In Vitro Peptide Permeation

**Figure 3:**
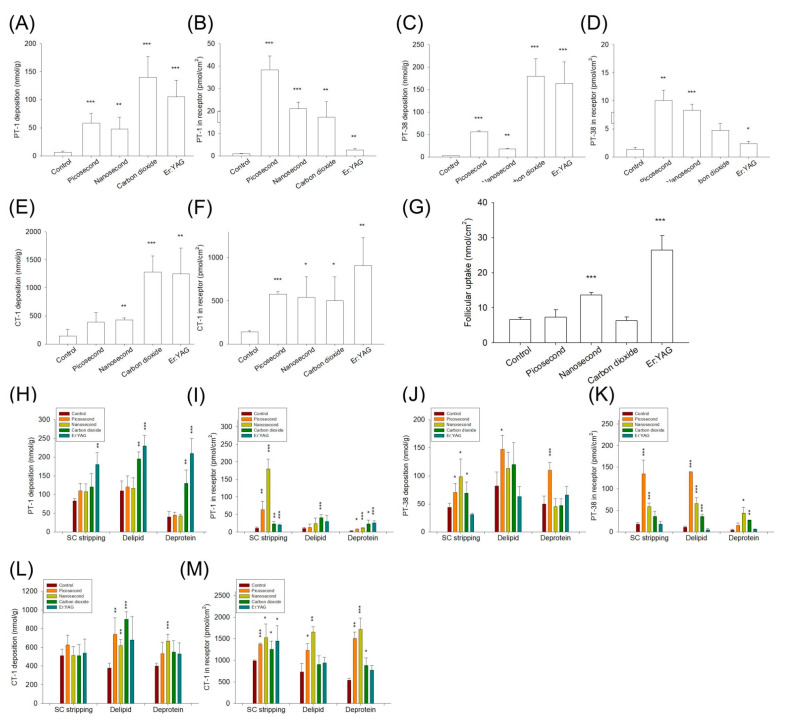
The in vitro pigskin deposition (nmol/g) and receptor amount (nmol/cm^2^) of the cosmeceutical peptides in the skin treated with and without laser treatment at 24 h: (**A**) skin deposition and (**B**) cumulative receptor amount of PT-1; (**C**) skin deposition and (**D**) cumulative receptor amount of PT-38; (**E**) skin deposition and (**F**) cumulative receptor amount of CT-1; (**G**) hair follicle uptake of PT-1; (**H**) skin deposition and (**I**) cumulative receptor amount of PT-1 via barrier-deficient skin; (**J**) skin deposition and (**K**) cumulative receptor amount of PT-38 via barrier-deficient skin; and (**L**) skin deposition and (**M**) cumulative receptor amount of CT-1 via barrier-deficient skin. The data are presented as the means of four experiments ± SEM. Note: * *p* < 0.05, ** *p* < 0.01, and *** *p* < 0.001 as compared to intact skin.

**Figure 4:**
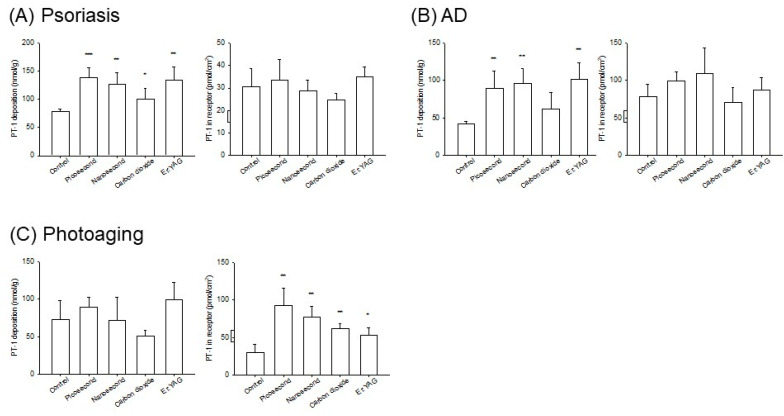
The in vitro mouse skin deposition (nmol/g) and receptor amount (nmol/cm^2^) of PT-1 in the inflamed skin with and without laser treatment at 24 h: (**A**) skin deposition and receptor amount of PT-1 via psoriasis-like skin; (**B**) skin deposition and receptor amount of PT-1 via AD-like skin; and (**C**) skin deposition and receptor amount of PT-1 via photoaged skin. The data are presented as the means of four experiments ± SEM. Note: * *p* < 0.05, ** *p* < 0.01, and *** *p* < 0.001 as compared to intact skin.

The pigskin deposition and PT-1, PT-38, and CT-1 amounts in the receptors of the in vitro Franz cell assembly were determined to evaluate the effect of the laser on skin delivery. Since baby pigskin is thinner than human skin, the peptides retained in the pigskin, and the receptors dictated the accumulation in the superficial layer and deeper skin strata, respectively. The comparison of the skin deposition and receptor accumulation of different peptides in different types of skin with or without a laser is shown in Figure 3 and Figure 4. The fold changes of the skin deposition and receptor accumulation of peptides by laser treatment compared with the untreated control are summarized in Table 2 and Table 3, as well as in Supplementary Tables S1–S3. The intracutaneous deposition of PT-1 in the intact skin was 6.5 nmol/g (Figure 3A). The ablative laser-enhanced PT-1 deposition was higher than that of the non-ablative Nd:YAG lasers. The picosecond Nd:YAG, nanosecond Nd:YAG, CO_2_, and Er:YAG lasers enhanced the PT-1 deposition by 9-, 7-, 22-, and 16-fold relative to the intact skin, respectively (Table 2). Contrary to the tendency for skin deposition, the non-ablative lasers showed a greater enhancement of the amount of PT-1 in the receptors than the ablative lasers (Figure 3B), and the picosecond Nd:YAG laser displayed the highest enhancement—by a factor of 40.

PT-38 demonstrated lower permeation in untreated skin compared to PT-1 (Figure 3C). The picosecond Nd:YAG, nanosecond Nd:YAG, microsecond CO_2_, and Er:YAG lasers increased the PT-38 deposition from 2.4 to 56.0, 17.8, 180.0, and 163.7 nmol/g, respectively. Similarly to PT-1, the non-ablative lasers increased the amount of PT-38 in receptors more than the ablative lasers (Figure 3D). The picosecond- and nanosecond-domain lasers increased the amount of PT-38 in the receptors, resulting in a seven- and six-fold greater level compared with the non-laser control. CT-1 showed the highest passive skin delivery among the three peptides (Figure 3E). The application of CT-1 onto the skin exposed to the ablative lasers resulted in a higher increase in the intracutaneous deposition compared to that exposed to the non-ablative lasers. A similar tendency was found for the CT-1 amount in the receptors (Figure 3F). The laser-assisted delivery of CT-1 resulted in a lesser increase as compared to the concentration of PT-1 and PT-38 in the receptors. The follicular accumulation of peptides in the skin after laser irradiation was also explored using PT-1 as the model permeant. The follicular PT-1 uptake after the nanosecond-domain and fully ablative Er:YAG laser treatment was two- and four-fold higher than that of the intact skin (Figure 3G), respectively, whereas the picosecond-domain and CO_2_ lasers did not improve the PT-1 transport to the hair follicles.

The PT-1 deposition was elevated from 6.5 to 82.6, 110.0, and 40.0 nmol/g by SC stripping, lipid removal, and protein denaturation (Figure 3H), respectively. Further Nd:YAG laser exposure of the barrier-deficient skin did not increase the PT-1 accumulation. The enhancement of PT-1 deposition on barrier-deficient skin was detectable in the cases where the ablative lasers were used. Although the non-ablative lasers could not promote PT-1 deposition in barrier-deficient skin, the picosecond and nanosecond modalities further increased the receptor accumulation by 19- and 53-fold compared with the SC-stripped skin without laser treatment (Figure 3I and Supplementary Table S1). This enhancement effect was lower for the ablative lasers. By contrast, compared with the non-ablative lasers, the ablative lasers contributed to a higher permeation enhancement on the delipidized and deproteinized skins. In contrast to PT-1, the PT-38 skin delivery was further enhanced by the non-ablative lasers as compared to the ablative lasers, with the picosecond modality showing the greatest enhancement (Figure 3J,K). The picosecond-domain laser-assisted PT-38 receptor penetration across the SC-stripped, delipidized, and deproteinized skin was 31-, 13-, and 2-fold higher than that across the skin without laser treatment (Supplementary Table S2). However, decreased permeation enhancement of the picosecond-domain laser was detected in the PT-38 deposition in the barrier-deficient skin when compared with the cumulative amount in the receptors. In general, the enhancement of the CT-1 deposition with different lasers was comparable (Figure 3L). The lasers did not largely facilitate CT-1 deposition inside the barrier-deficient skin. The non-ablative lasers exerted a greater enhancement of the CT-1 amount in the receptors on the delipidized and deproteinized skins compared to the ablative lasers (Figure 3M). The amount of CT-1 in the receptors across the SC-stripped skin enhanced with all lasers was limited (approximately 1.5-fold) and comparable (Supplementary Table S3).

The cosmeceutical peptides represented some activities that could suppress cutaneous inflammation. In the psoriasiform skin, PT-1 deposition was enhanced with all lasers compared to the control group at 1.3−1.8 times (Table 3 and Figure 4A, left panel). The lasers were unable to enhance the accumulation of PT-1 in the receptors through the psoriasiform skin (Figure 4A, right panel). This effect was also observed in the AD-like skin (Figure 4B, right panel). The fractionated CO_2_ laser also did not enhance intracutaneous PT-1 deposition after treatment of the AD-like skin (Figure 4B, left panel). The Nd:YAG and Er:YAG lasers led to a similar increase in PT-1 deposition in the AD-like skin (2.1−2.4-fold). Compared with the psoriasis- and AD-like skins, the lasers could promote the amount of PT-1 in the receptors in the photoaged skin but not intracutaneous accumulation (Figure 4C). The picosecond-domain laser showed the greatest enhancement of the cumulative PT-1 amount in the receptors in the photoaged skin compared with the non-laser treatment (3.1-fold), followed by the nanosecond (2.5-fold), CO_2_ (2.0-fold), and Er:YAG laser (1.7-fold) treatments.

### 3.4. Peptide Distribution in the Skin

**Figure 5:**
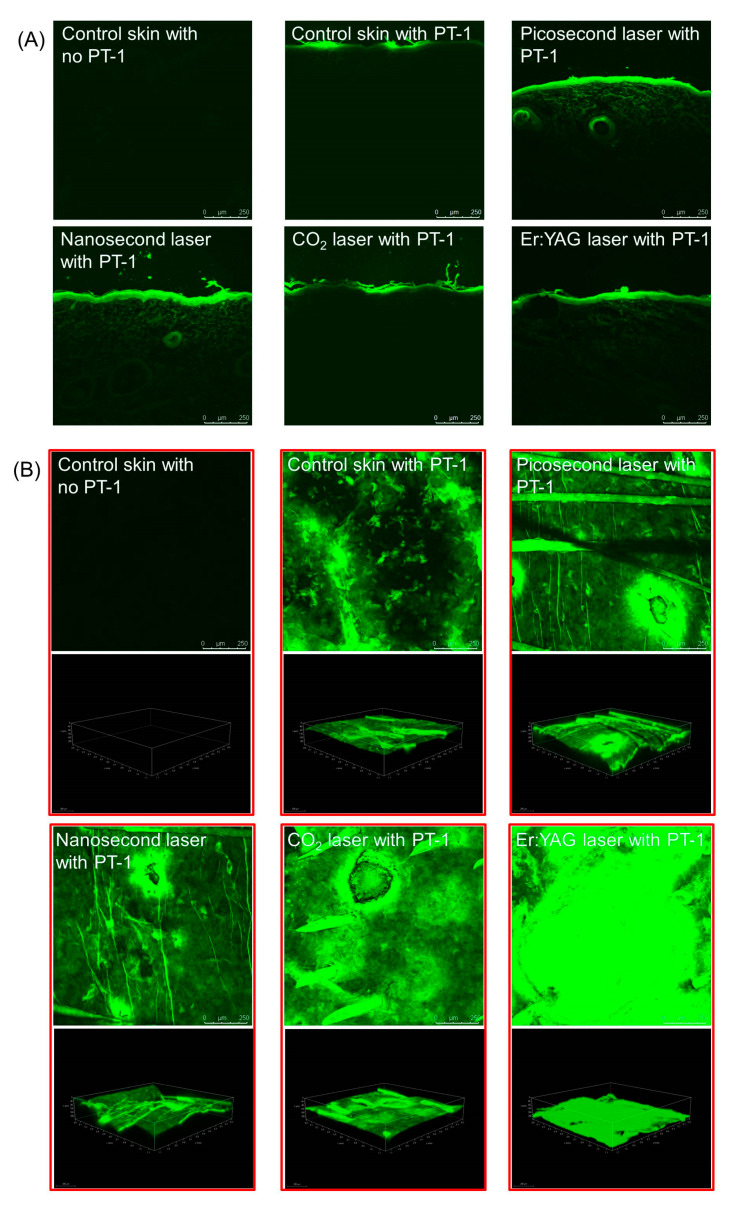
The biodistribution of FITC-conjugated PT-1 in the pigskin treated with and without laser treatment: (**A**) the skin observed by fluorescence microscopy in a vertical view; and (**B**) the skin observed by confocal laser scanning microscopy in a horizontal view. The upper panel is 2D (*xy*-axis) imaging of the skin. The lower panel is 3D (xyz-axis) imaging of the skin.

Following the in vitro experiment with the FITC-labeled peptides, the skin was imaged to visualize the PT-1 distribution throughout the skin. The vertical view of the PT-1 skin distribution was displayed under fluorescence microscopy. Fluorescence was not detected in the control skin using normal saline treatment only (Figure 5A, upper left panel), indicating that there was no autosignal from the skin. After topical PT-1 delivery, some fluorescence signals were observed in the superficial layer of the intact skin (Figure 5A, upper middle panel). PT-1 fluorescence was shown beyond the epidermis and into the dermis after the picosecond- and nanosecond-domain laser treatments (Figure 5A, upper right and lower left panels, respectively). By contrast, fluorescence was only seen in the epidermal tissue of the pigskin subjected to ablative laser application (Figure 5A, lower middle and lower right panels). The fluorescence in the Er:YAG laser-irradiated skin was distributed in the epidermis in a continuous manner. The distribution of the FITC-labeled PT-1 peptide was realized by confocal microscopy in the horizontal view. Both the 2D (xy-plane) and 3D (xyz-plane) views of the summarized 15 fragments of skin were established. No autofluorescence was shown for the blank skin without PT-1 intervention in either the 2D or the 3D images (Figure 5B, upper left panel). The FITC fluorescence was distributed in the superficial layer of the intact skin (Figure 5B, upper middle panel). The 3D view demonstrated the ability of the non-ablative lasers to deliver PT-1 to the deeper skin layer (Figure 3B, upper right and lower left panels). The fluorescence from PT-1 was especially present in and around the microdots created by the fractional Nd:YAG laser. The PT-1 distribution surrounding the dots manifested a lateral diffusion from the microdots. The PT-1 followed the microchannels created by the fractional CO_2_ laser for transport into the skin (Figure 5B, lower middle panel). The fluorescence-stained circles exhibited a diameter of approximately 350 nm. There was radial diffusion of PT-1 from the microchannels. The confocal image displayed a strong and homogeneous PT-1 distribution in the superficial skin irradiated by the Er:YAG laser (Figure 5B, lower right panel).

### 3.5. In Vivo Skin Deposition of PT-1

**Figure 6:**
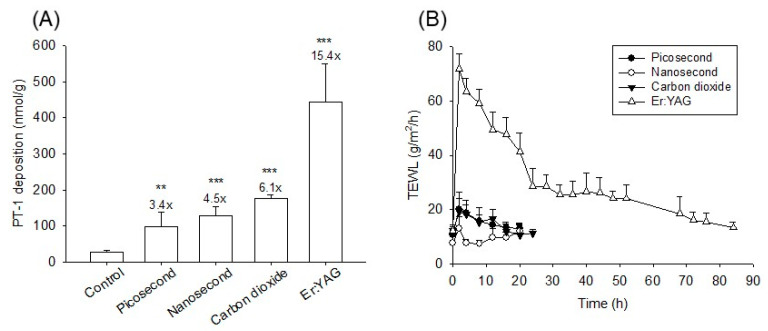
The in vivo nude mouse skin deposition (nmol/g) of PT-1 at 6 h and the barrier function recovery with and without laser treatment: (**A**) skin deposition of PT-1; and (**B**) skin barrier function determined by TEWL. The data are presented as the means of six experiments ± SEM. Note: ** *p* < 0.01 and *** *p* < 0.001 as compared to intact skin.

This study next evaluated the in vivo PT-1 deposition in the nude mouse skin. Each mouse received laser illumination on the dorsal skin. The PT-1 deposition was 28.8 nmol/g in the intact skin, which was higher than that in the in vitro pigskin (6.5 nmol/g) (Figure 6A). Similar to the in vitro pigskin deposition, the ablative lasers were more efficacious than the non-ablative lasers at improving PT-1 delivery. The fully ablative Er:YAG laser offered the greatest enhancement of intracutaneous PT-1 deposition (15-fold enhancement). The picosecond- and nanosecond-domain lasers, meanwhile, enhanced PT-1 deposition three- and five-fold compared with the control, respectively.

### 3.6. In Vivo Skin Barrier Function Recovery

Once the efficacy of the laser-assisted peptide delivery was confirmed, the skin barrier was recovered after laser exposure and their effect was assessed in terms of safety. The skin barrier integrity was measured by TEWL on the nude mouse skin using a Tewameter. The TEWL baseline before laser treatment was approximately 10 g/m^2^/h (Figure 6B). The skin integrity detected by TEWL was compromised by the lasers immediately after treatment. The TEWL levels of the skin treated by the picosecond, nanosecond, CO_2_, and Er:YAG lasers after two hours were 20, 13, 19, and 71 g/m^2^/h, respectively. The TEWL level returned to the baseline after four hours for the nanosecond-domain laser intervention. The picosecond and CO_2_ modalities showed a similar tendency of barrier function recovery, with complete recovery at 16 h. With the fully ablative laser, a longer period of 76−84 h was needed to recover an intact barrier function.

## 4. Discussion

Topical peptides are limited in their cosmeceutical applications due to insufficient skin absorption. To improve this drawback, we investigated the use of picosecond- and nanosecond-domain laser-assisted skin delivery for cosmetic peptide transport. The results of this study suggest the beneficial effect of topical peptides facilitated by non-ablative Nd:YAG lasers. The peptide permeation enhancement by the Nd:YAG laser could reach the level promoted by the microsecond-domain ablative lasers. In this study, a faster recovery of the skin barrier function was achieved with the Nd:YAG laser compared to that with the ablative lasers.

Although the one-week-old pigs had a relatively thin skin layer compared to the skin of humans and adult pigs, it was an ideal permeation barrier model for mounting the Franz cell between the donor and the receptor. For topical delivery purposes, the cumulative amount of the permeants in the receptors can be an indicator of the delivery to deeper skin strata because of the thin skin layer of baby pigs [20,21]. Receptor accumulation can be a marker of deeper skin layer permeation when using dermatomed human skin with a thickness < 0.5 mm [22]. The pigskin thickness used here was about 0.55 mm, simulating the dermatomed human skin used in the previous study. Significant thermal coagulation around the micropores was observed in the fractional CO_2_ laser-treated pigskin, while this coagulation was not observed in the Nd:YAG laser-treated skin. Microsecond-domain lasers usually have considerable photocoagulation and photoacoustic effects. On the contrary, picosecond- and nanosecond-domain modalities offer a nearly complete photoacoustic effect, leading to the minimal transfer of heat to the tissue [23]. The non-ablative Nd:YAG laser caused mild vacuolation in the upper epidermis, while the SC and the dermis remained intact. Nd:YAG lasers can generate a micro-injury zone that presents histologically as intraepidermal vacuoles [24]. The picosecond- and nanosecond-domain laser energy transferred to the skin tissue can rapidly generate an increase in the temperature of the tissue and water and evoke cavitation to create vacuoles [25]. The fluence of the 1064 nm focused picosecond-domain lasers used in clinical treatment usually causes large vacuoles in both the epidermis and the dermis [26]. The fluence (1.4 J/cm^2^) used in this study only produced vacuoles in the upper epidermis, indicating a mild disruption to the skin structure. The resulting CO_2_ laser-created microdots were smaller than the microbeam. In addition to the thermal coagulation formation by the microbeam, a quick contraction of the CO_2_ laser-generated pores soon after their formation made it possible to create small micropores [27]. We observed some disruptions on the Nd:YAG laser-treated skin surface in the SEM image, although this change was not detectable under optical microscopy. The Nd:YAG laser at 1064 nm made it possible to disorganize corneocytes in the SC without ablation [28].

This study showed that the passive permeability of CT-1 was significantly higher than that of the messenger peptides PT-1 and PT-38. The lipophilicity of permeants is a vital determinant of skin absorption [29], and higher lipophilicity of the permeant is associated with higher skin diffusion because of the facile partitioning into the lipophilic SC lipids. Although PT-1 and PT-38 manifested higher lipophilicity than CT-1, this study found that their diffusion via intact skin was limited. In addition to the partition coefficient, the total polarity surface contributed to the molecular estimation of lipophilicity. The total polarity surface is inversely correlated with the transport across the skin [30]. The total polarity surface of both messenger peptides was higher than that of CT-1. As PT-1 and PT-38 contain many amide bonds such as hydrogen bond acceptor and donor groups, the cutaneous transport of peptides with large numbers of hydrogen bonds may be low because of the increase in molecular polarity [31]. The total polarity surface and hydrogen bond could not fully elucidate the facile penetration of CT-1 into the skin. The molecular size of the permeant is important to regulate skin absorption and can be rated by the MW and MV. Given the barrier function of intact skin, the MW of topical peptides should be <500 Da so that they can pass through the barrier [1]. CT-1 (but not PT-1 or PT-38) fits this criterion. MV is a parameter used to estimate the molecular size considering both the MW and the steric structure of a molecule [32]. The MV of CT-1 was also much smaller than those of PT-1 and PT-38. The long-chain palmitoyl moiety of PT-1 and PT-38 may cause a steric hindrance when passing across the skin.

A previous study [33] suggested that CT-1 penetrates the skin rapidly to deliver minerals to the skin. Copper assists with the permeation of CT-1 as it can rebind to endogenous amino acids with high nucleophilic donor capacity in the skin [34]. PT-1 and PT-38 show similar physicochemical properties. Our data showed that PT-1 had a higher intact skin deposition than PT-38, whereas the cumulative amount in the receptors manifested a contrary tendency. Although the Alog *P* of PT-38 (2.42) was slightly greater than that of PT-1 (2.27), the greater hydrogen bond number and total polarity surface of PT-38 could cause the unfavorable retention in lipophilic SC. Thus, PT-38 could bypass the SC and enter viable skin, resulting in a facile diffusion into the receptor compartment. The laser treatment increased the PT-38 skin deposition more so than that of PT-1, signifying that the laser could conquer the SC barrier to enhance PT-38 residence in the skin reservoir. It should be noted that the peptides used in this study were conjugated with FITC for the aim of quantitative detection and fluorescent microscopic observation. The skin permeation of FITC-conjugated peptides might be slightly higher than that of unconjugated peptides because of the high log *P* of FITC (about 3.1) for facile delivery or partitioning into the SC. On the other hand, the increased molecular size due to the additional FITC moiety could decrease the delivery to achieve an offset effect on skin absorption. The difference of skin delivery between the conjugated and unconjugated molecules might be small, based on the case of insulin as described in a previous study [35], which found the mono conjugation of FITC to insulin only slightly increased the permeation across the biomembrane (kidney cell monolayer). The tendency of skin delivery for peptides could be similar for both conjugated and unconjugated molecules since the effect accompanied with FITC was similar for all the peptides tested. The barrier-deficient skin model enabled the investigation of the permeation pathways for the peptides. The outermost SC was the predominant delivery barrier of the three peptides because of the significantly increased absorption after SC stripping. The permeation of PT-1, PT-38, and CT-1 in the delipidized skin nearly reached that of the SC-stripped skin, indicating that the cosmetic peptides mainly partitioned into the lipid domain of the SC for subsequent transport. The intracellular protein route played a minor role in the intercellular lipid pathway for the topical application of peptides.

In addition to the SC layer, the viable epidermis/dermis represented a significant diffusion barrier, as the lasers could further increase peptide absorption via the SC-stripped skin. The fractional CO_2_ laser could largely promote the permeation of the peptides through the skin. For instance, the CO_2_ laser elevated the skin deposition and cumulative PT-1 in the receptors 22-fold compared with the untreated skin. The confocal image showed that the CO_2_ laser created microchannels that formed depots for PT-1 accumulation, followed by lateral transport to elicit extensive distribution in the skin. The CO_2_ laser induced micropores through controlled ablation of the superficial skin layer. This aqueous channel bypassed the unfavorable SC barrier and reduced the penetration path length. The fractional laser can create microchannels to increase both the total area of effective permeation and the reservoir for delivery [36]. On the contrary, the non-ablative Nd:YAG modality could magnify the peptide permeation into and across the pigskin. Some vacuoles were found in the epidermis of the Nd:YAG laser-treated skin. The 1064 nm laser could drive the shockwave at a supersonic velocity to create cavitation, which generated a microscopic vacuolar tissue reaction [24]. In addition to vacuole formation, this shockwave temporally disrupted the SC structure to enhance cutaneous drug delivery [22]. Our SEM image depicted ultrastructural changes in the skin surface after Nd:YAG laser exposure. The disorganized SC permitted the transport of peptides into the skin. A significant radial diffusion of PT-1 from the microdots created by the Nd:YAG laser was detected by confocal imaging. This lateral diffusion was greater than that of the CO_2_ laser-induced microchannels and could be due to the establishment of thermal coagulation around the MTZ by the CO_2_ laser. The thick coagulation restricted permeant passage across the microchannels [37]. The fully ablative Er:YAG laser at 2940 nm was effective for controlled peeling of the SC with minimal thermal effect according to the histological examination. The SC removal by the Er:YAG laser reduced the diffusion path length and permeation barrier property to allow increased drug absorption [21]. In this study, the significant increase in TEWL after Er:YAG laser ablation demonstrated a compromised barrier function to expand peptide delivery.

The in vitro skin absorption was calculated as the sum of the quantity deposited and penetrated. The cumulative amount of receptors could be recognized as an accumulation in the deeper skin strata (>150 μm skin layer) [22]. The skin deposition could be regarded as superficial skin accumulation owing to the use of baby pigskin. The increased peptide delivery into the receptors by the lasers equated to a greater concentration of peptides within the deeper skin layers. The in vitro peptide absorption result demonstrated that the non-ablative lasers chiefly increased the number of peptides in the receptors to a higher level relative to the pigskin deposition. An antagonistic tendency was found for the ablative lasers. The confocal microscopic result showed a facile delivery of PT-1 into the dermis after Nd:YAG laser treatment. The accumulation of peptides in the hydrophilic dermis allowed for the subsequent transport into the receptors. The shockwave generated by the lasers was confirmed to deliver the drugs into the deeper strata [19]. For the treatment of aged skin, a deeper diffusion of cosmeceutical peptides is needed to activate the synthesis of collagen and elastin in the dermis. Topical peptides usually cannot reach the dermis for efficient anti-aging interventions [38]. With picosecond- and nanosecond-domain Nd:YAG laser treatment, dermal delivery of the peptides was greatly increased; thus, preventive or therapeutic concentrations of the peptides within the target site were reached. The comparison of different peptides demonstrated that the laser-assisted messenger peptides (PT-1 and PT-38) had greater absorption enhancement than the carrier peptide (CT-1). This result signified the greater selectivity of palmitoyl peptides in diffusing through laser-treated skin.

Cosmeceutical peptides such as PT-1 and CT-1 have been reported to initiate anti-inflammatory activities for mitigating skin irritation and accelerating wound healing [34,39]. Laser-facilitated delivery can be utilized to increase the anti-inflammatory capacity of topical peptides. More evidence is required to understand the potential application of laser-assisted delivery to cutaneous diseases. We developed psoriasis-like, AD-like, and photoaged skins in mice to analyze peptide delivery through inflamed skin. These inflammatory models were established in our previous reports and established a similar characteristic of histopathology with inflamed human skin [17,30,40,41]. All laser modalities enhanced the deposition of PT-1 into the psoriasiform and AD-like skins at a comparable level. The enhancement was less than that of healthy skin because of the compromised barrier of the inflamed skin. The inflamed skin reservoir was saturated by the topically applied peptides; thus, further laser treatment showed a finite ability to elevate the saturation level of peptides in the skin. The lasers increased the PT-1 amount in the receptors but not the intracutaneous deposition in the photoaged skin. The penetration enhancement of the non-ablative lasers was greater than that of the ablative lasers. It is beneficial for Nd:YAG laser-assisted peptide delivery to arrest photoaged inflammation since UVA largely induces collagen and elastin fragmentation in the dermis.

The barrier-deficient pigskin was also valuable for defining the effect of laser-assisted delivery on diseased skin. SC stripping can be a robust model of dermatitis [9]. Delipidized skin can be used as a model correlating with psoriasis, ichthyosis, and xerosis [42]. SC protein denaturation mimics atopic dermatitis and photoaging [43]. These barrier-deficient models offer more reproducible and simpler processes to produce damaged skin. Similar to the results for inflamed skin, the lasers demonstrated the inferior enhancement of laser-mediated peptide delivery in the barrier-deficient skin compared to the intact skin. However, the non-ablative lasers were sufficiently powerful to promote the amount of PT-1 and PT-38 in the receptors in the barrier-deficient skin. Cosmetic peptides can also be applied in hair care. Cosmeceutical peptides have a high affinity to hair keratin, which improves the strength and elasticity of hair [44]. Our results suggest that nanosecond-domain lasers and Er:YAG lasers are effective for upgrading peptide accumulation in hair follicles. The influence of Er:YAG lasers on increasing the follicular targeting of antialopecia drugs was confirmed in a previous study [19]. The Er:YAG laser propagated ablation and photomechanical waves around the follicles. The laser also unfolded closed follicles that were covered with sebum, cell debris, and desquamated corneocytes. The shockwave induced by the Nd:YAG laser may have had a similar effect on raising the follicular delivery, although this effect was only observed for the nanosecond-domain laser. The follicular pathway also provides a reservoir for the delivery of permeants into deeper skin layers [45], which can indicate the high delivery of peptides to the receptors following Nd:YAG laser exposure.

The in vivo nude mouse skin deposition was evaluated by the topical administration of PT-1 at 150 μM. This dose was lower than the safe dose for guinea pigs and humans in a previous skin irritation study [46]. The ablative lasers demonstrated greater enhancement of the in vitro PT-1 deposition compared with the non-ablative lasers. Nude mouse skin is thinner than pig and human skin. In vivo, PT-1 deposition can be considered superficial skin accumulation. The in vivo result supported the notion that ablative laser-assisted delivery could more effectively facilitate the superficial uptake of peptides. Laser-assisted drug delivery may be harmful because of the potential for bacterial invasion across disrupted skin and possible systemic drug absorption. Quick recovery of intact skin is important to verify the safe use of laser-assisted delivery. Our TEWL result revealed that a prolonged healing time was necessary after Er:YAG laser illumination on nude mouse skin. This disadvantage could be improved by using a fractionated strategy. The intact skin area around the micropores accelerated the healing of the laser-treated damage and shortened the recovery duration. In this study, the laser fluence was much lower than that used for skin rejuvenation or pigmentation removal in the clinic. The skin barrier function was recovered within 16 h for the fractional picosecond-domain and CO_2_ lasers. The skin treated by the nanosecond-domain lasers could even recover to normal status within 4 h. Although the Nd:YAG laser-induced SC breakdown and epidermal vacuolation compromised the barrier feature, these disruptions could be repaired within a short period to recover the normal condition.

The picosecond and nanosecond modalities generally exhibited similar peptide delivery enhancements according to the in vitro skin permeation, peptide distribution in the skin, and in vivo intracutaneous deposition. On the contrary, the nanosecond (but not picosecond) laser could augment the follicular peptide uptake. Faster recovery of the barrier function was detected for skin irradiated by the nanosecond-domain laser compared to that irradiated by the picosecond-domain laser. The nanosecond modality could be a better choice than the picosecond domain. Although ablative lasers can successfully deliver peptides to the superficial skin layer, superficial resurfacing may contribute to skin irritation and bacterial infection [47,48]. The non-ablative Nd:YAG laser was both effective and safe for promoting peptide delivery. This may allow for more practical methods of prevention or treatment using topical peptides and potentially reduce treatment duration, side effects, and peptide doses.

## 5. Conclusions

Currently, several peptides are added to cosmetic or pharmaceutical products. Topical peptides have the potential to prevent or treat skin aging and inflammation. The skin delivery of peptides should be ameliorated to increase their application in the cosmeceutical field. Herein, we employed laser-mediated delivery of topical peptides. Both non-ablative and ablative lasers were used in this study. The non-ablative Nd:YAG laser with picosecond and nanosecond domains notably increased the skin absorption of peptides and induced a more profound delivery to the receptor compartment of the Franz cell. The non-ablative laser delivered peptides to the dermis, as visualized under fluorescence microscopy. The lasers also promoted peptide permeation via barrier-deficient or inflamed skin, although this enhancement was inferior to that via intact skin. The treatment by the nanosecond-domain Nd:YAG laser showed the fastest skin barrier function recovery, followed by that obtained with the picosecond, CO_2_, and Er:YAG lasers. The nanosecond-domain laser was expected to better perform laser-assisted peptide delivery because of its capability to enhance delivery to deeper skin strata and hair follicles and its ability to minimally impair barrier properties.

## Figures and Tables

**Table 1:** Physicochemical properties of cosmetic peptides.

Compound	MW ^a^ (Da)	MV ^b^	Alog *P* ^c^	Hydrogen Bond Acceptor Number	Hydrogen Bond Donor Number	Total Polarity Surface
PT-1	577.80	513.47	2.27	8	4	658.11
PT-38	604.84	526.16	2.42	9	5	699.42
CT-1	344.41	284.68	–2.08	7	6	400.50

^a^ MW, molecular weight; ^b^ MV, molecular volume; ^c^ Alog *P*, partition coefficient measured by molecular modeling.

**Table 2:** The fold change of the mean value of skin deposition and cumulative amounts in the receptors after laser treatment of intact pigskin as compared with nontreatment control.

	Peptide	Picosecond Nd:YAG	Nanosecond Nd:YAG	CO_2_	Er:YAG
Skin deposition	PT-1	9.0	7.4	21.6	16.2
	PT-38	23.7	7.6	76.3	69.3
	CT-1	2.8	3.1	9.1	8.9
Amount in the receptors	PT-1	40.4	22.1	18.2	2.8
	PT-38	7.4	6.1	3.5	1.7
	CT-1	4.1	3.9	3.6	6.5

**Table 3:** The fold change of the mean value of PT-1 skin deposition and cumulative amount in the receptors after laser treatment as compared with nontreatment control on inflamed skin.

	Inflamed Skin Type	Picosecond Nd:YAG	Nanosecond Nd:YAG	CO_2_	Er:YAG
Skin deposition	Psoriasis	1.8	1.6	1.3	1.7
	AD	2.1	2.3	1.5	2.4
	Photoaging	1.2	1.0	0.7	1.4
Amount in the receptors	Psoriasis	1.1	0.9	0.8	1.1
	AD	1.3	1.4	0.9	1.1
	Photoaging	3.1	2.5	2.0	1.7

## Supplementary Materials

The following supporting information can be downloaded at: https://www.mdpi.com/article/10.3390/pharmaceutics14020450/s1. Table S1. The fold change of mean value of PT-1 skin deposition and cumulative amount in receptor after laser treatment as compared with nontreatment control on barrier-deficient skins, Table S2. The fold change of mean value of PT-38 skin deposition and cumulative amount in receptor after laser treatment as compared with nontreatment control on barrier-deficient skins, Table S3. The fold change of mean value of CT-1 skin deposition and cumulative amount in receptor after laser treatment as compared with nontreatment control on barrier-deficient skins.
