# Supplementary material for: Cutaneous Delivery of Cosmeceutical Peptides Enhanced by Picosecond- and Nanosecond-Domain Nd:YAG Lasers with Quick Recovery of the Skin Barrier Function: Comparison with Microsecond-Domain Ablative Lasers
Source: Pharmaceutics. 2022 Feb 19;14(2):450. doi: 10.3390/pharmaceutics14020450 (PMC8880571; doi:10.3390/pharmaceutics14020450)
Supplement: Supplementary file 1 [file pharmaceutics-14-00450-s001.zip › pharmaceutics-1573550-supplementary.pdf]

pharmaceutics

Supplementary Materials: Cutaneous Delivery of Cosmeceutical Peptides Enhanced by Picosecond- and Nanosecond-Domain Nd:YAG Lasers with Quick Recovery of the Skin Barrier Function: Comparison with Microsecond-Domain Ablative Lasers

Woan-Ruoh Lee, Chien-Yu Hsiao, Zi-Yu Chang, Pei-Wen Wang, Ibrahim A. Aljuffali, Jie-Yu Lin and Jia-You Fang

Table S1. The fold change of mean value of PT-1 skin deposition and cumulative amount in receptor after laser treatment as compared with nontreatment control on barrier-deficient skins.

	**Barrier-deficient skin**	**Picosecond Nd:YAG**	**Nanosecond Nd:YAG**	**CO_2**	**Er:YAG**
Skin deposition	SC-stripping	1.3	1.3	1.5	2.2
	Delipid	1.1	1.1	1.8	2.1
	Deprotein	1.1	1.1	3.3	5.3
Amount in receptor	SC-stripping	6.1	17.3	2.2	1.9
	Delipid	1.3	2.4	4.1	3.0
	Deprotein	2.5	4.1	8.5	9.6

Table S2. The fold change of mean value of PT-38 skin deposition and cumulative amount in receptor after laser treatment as compared with nontreatment control on barrier-deficient skins.

	**Barrier-deficient skin**	**Picosecond Nd:YAG**	**Nanosecond Nd:YAG**	**CO_2**	**Er:YAG**
Skin deposition	SC-stripping	1.6	2.2	1.6	0.7
	Delipid	1.8	1.4	1.5	0.8
	Deprotein	2.2	0.9	0.9	1.3
Amount in receptor	SC-stripping	7.5	3.3	2.0	1.0
	Delipid	12.5	6.0	3.3	0.5
	Deprotein	3.5	9.9	6.1	1.3

Table S3. The fold change of mean value of CT-1 skin deposition and cumulative amount in receptor after laser treatment as compared with nontreatment control on barrier-deficient skins.

	**Barrier-deficient skin**	**Picosecond Nd:YAG**	**Nanosecond Nd:YAG**	**CO_2**	**Er:YAG**
Skin deposition	SC-stripping	1.2	1.0	1.0	1.1
	Delipid	1.9	1.6	2.4	1.8
	Deprotein	1.3	1.7	1.4	1.3
Amount in receptor	SC-stripping	1.4	1.5	1.3	1.5
	Delipid	1.7	2.3	1.2	1.3
	Deprotein	2.8	3.2	1.6	1.4
